# The developing landscape of combinatorial therapies of immune checkpoint blockade with DNA damage repair inhibitors for the treatment of breast and ovarian cancers

**DOI:** 10.1186/s13045-021-01218-8

**Published:** 2021-12-20

**Authors:** Lingling Zhu, Jiewei Liu, Jiang Chen, Qinghua Zhou

**Affiliations:** 1 Lung Cancer Center, West China Hospital of Sichuan University, Chengdu, 610041 Sichuan Province China; 2 Department of General Surgery, Sir Run Run Shaw Hospital, Zhejiang University, Hangzhou, 310016 Zhejiang Province China

**Keywords:** Immune checkpoint blockade, Breast cancer, Ovarian cancer, DNA damage repair inhibitor

## Abstract

The use of immune checkpoint blockade (ICB) using antibodies against programmed death receptor (PD)-1, PD ligand (PD-L)-1, and cytotoxic T-lymphocyte antigen 4 (CTLA-4) has redefined the therapeutic landscape in solid tumors, including skin, lung, bladder, liver, renal, and breast tumors. However, overall response rates to ICB therapy remain limited in PD-L1-negative patients. Thus, rational and effective combination therapies will be needed to address ICB treatment resistance in these patients, as well as in PD-L1-positive patients who have progressed under ICB treatment. DNA damage repair inhibitors (DDRis) may activate T-cell responses and trigger inflammatory cytokines release and eventually immunogenic cancer cell death by amplifying DNA damage and generating immunogenic neoantigens, especially in DDR-defective tumors. DDRi may also lead to adaptive PD-L1 upregulation, providing a rationale for PD-L1/PD-1 blockade. Thus, based on preclinical evidence of efficacy and no significant overlapping toxicity, some ICB/DDRi combinations have rapidly progressed to clinical testing in breast and ovarian cancers. Here, we summarize the available clinical data on the combination of ICB with DDRi agents for treating breast and ovarian cancers and discuss the mechanisms of action and other lessons learned from translational studies conducted to date. We also review potential biomarkers to select patients most likely to respond to ICB/DDRi combination therapy.

**Supplementary Information:**

The online version contains supplementary material available at 10.1186/s13045-021-01218-8.

## Background

Remarkable progress has been made in the clinical application of cancer immunotherapies harnessing the immune system to identify and eradicate breast and ovarian tumors. The most notable example is the emergence of immune checkpoint blockade (ICB) to inhibit negative regulators of effector T-cell-mediated immunity. However, when administered alone, ICB approaches, such as anti-cytotoxic T-lymphocyte-associated protein 4 (CTLA-4), anti-programmed cell death protein 1 (PD-1), and/or anti-programmed death-ligand 1 (PD-L1) antibodies, generally elicit low objective response rates (ORRs), ranging from 0 to 33%, which are durable only in a minority of cancer patients [1–3]. For example, ORRs for ICB monotherapy range from 18.5 to 39.4% in the most frequent malignancy in women, breast cancer (BC) [4], and from 8% to 9.6% in ovarian cancer (OC) [5]. To address this unmet need, and improve the efficacy of ICB, multiple combinatorial strategies are currently being developed, some of which include DNA damage response (DDR) inhibitors (DDRis).

Defects in DDR genes hold the potential to function as biomarkers of response to ICB across multiple types of tumors [6]. The rationale for combining ICB with DDRi is also based on several studies that have demonstrated the critical role of DDR in the efficacy of cancer immunotherapy [7]. DDR proteins protect the integrity of the genome following DNA damage caused by endogenous factors (e.g., reactive oxygen species and errors during DNA replication) and exogenous insults (e.g., ultraviolet radiation, smoking, chemical substances) [8]. Thus, the use of DDRi might augment the tumor mutational burden (TMB), thereby increasing neoantigen production and anticancer T-cell activity [9], potentiating antitumor immunity. For example, targeting poly-ADP-ribose polymerase (PARP) markedly increased PD-L1 expression in cancer cells, augmenting the antitumor effect of PD-L1 blockade and cytotoxic T-cell infiltration in gynecologic cancers [10]. Therefore, patients with tumors carrying DNA mismatch repair (MMR) deficiency (MMRd), homologous recombination deficiency (HRD), breast cancer 1/2 (BRCA1/2) genetic defects, or other defects in DDR genes [9], who present more TMB and neoantigens, can benefit from DDRi [11]. For example, MMRd is characterized by the loss of function of the MMR pathway and can generate many insertion and deletion (indel) mutations; this indel mutational load has generated a substantial number of immunogenic neoantigens, potentially driving immunotherapeutic responses [12]. Moreover, compared to homologous recombination repair (HRR), a conservative mechanism contributing to DNA double-strand break (DSB) repair, HRD, such as BRCA-mutation in tumors, enhanced neoantigen burden [13].

Of note, deficiency of DDR genes occurs in a wide variety of malignancies, such as prostate, bladder, pancreatic, non-small cell lung cancers, and triple-negative BC (TNBC) [11]. HRD occurred in more than 20% of BCs, as well as OCs, pancreatic cancers, and gastric cancers [14] and in approximately 50% of epithelial OCs [15] and 69% of TNBC [16]. The majority of OCs and BCs are originating from epithelial cells that undergo constant division and cyclical exposure to estrogen during the female hormonal cycle, making them vulnerable to DNA damage [17]. According to the concept of a synthetic lethality among a functional genetic defect in an HR-related gene, the combination of DDRi and ICB in BCs and OCs with HRD is promising. For instance, DDRis, namely PARP inhibitors (PARPi) [18] and checkpoint kinase 1 (CHK1) inhibitor [19], have predominantly been studied in patients with breast or ovarian cancers. Still, the interactions between ICB and DDR pathways vary, and the synergy between DDRi and ICB against cancers, independent of DDR deficiency status, needs to be further clarified according to the latest preclinical models and clinical data. For instance, in both HRD and HRR settings, clinical evidence for OCs showed a synergistic antitumor activity of PARPi in combination with ICBs [5].

Besides PARPi, other agents and factors targeting the proteins involved in the DDR pathway include ataxia telangiectasia-mutated (ATM), CHK1, ataxia telangiectasia and Rad3-related protein (ATR), DNA-dependent protein kinase (DNA-PK), WEE1, classical non-homologous end joining (cNHEJ), and alternative end joining (Alt EJ) [9, 20]. Of the solid tumors reviewed, OCs have demonstrated efficacy in the treatment of DDRi. Moreover, OC is the first cause of death from and the second most common gynecological malignancy [21]. To date, three PARPi, olaparib, rucaparib, and niraparib, are approved by FDA for treatment of OCs. Among them, rucaparib [22, 23] is approved in the relapsed setting for OC patients with BRCA mutations, olaparib for first-line maintenance treatment in newly diagnosed stage III-IV ovarian patients who are in complete or partial synergistic antitumor activity of PARP inhibitors (PARPi) in combination with ICBs [5]. response to first-line platinum-based chemotherapy or to first-line chemotherapy [24] plus bevacizumab combination [25], and olaparib [26, 27], rucaparib [28] as well as niraparib [29] for maintenance therapy in patients with platinum sensitive recurrent OCs (after ≥ 2 lines chemotherapy), regardless of BRCA status (Table 1).

**Table 1:** FDA-approved DDR inhibitors in ovarian cancers in the past 5 years (2016–2021)

PARP inhibitors	Trial number	Disease setting	FDA approval	Approved mutation status	Study phase	Approved year
Olaparib	NCT01874353	Recurrent BRCA1/2-mutant ovarian cancer with PR or CR to most recent line of platinum-based chemotherapy (after ≥ 2 lines of chemotherapy)	Maintenance therapy for patients with advanced stage ovarian cancer in CR or PR after platinum-based chemotherapy	Irrespective of BRCA1/2 status	III	2017
	NCT00753545	Recurrent ovarian cancer with a PR or CR to most recent line of platinum-based chemotherapy (after ≥ 2 lines chemotherapy)	Maintenance therapy in women with advanced stage ovarian cancer in CR or PR after platinum-based chemotherapy	Irrespective of BRCA1/2 status	II	2017
	NCT01844986	Newly diagnosed, stage III-IV ovarian cancer with BRCA mutation	First-line maintenance treatment of adult patients with stage III-IV ovarian, cancer who are in CR or PR to first-line platinum-based chemotherapy	Deleterious or suspected deleterious g/sBRCA-mutations	III	2018
	NCT02477644, NCT03737643	Newly diagnosed, stage III-IV OC (other histologies if gBRCAm) with CR or PR to standard platinum based chemotherapy given with bevacizumab	First-line maintenance treatment of patients with stage III-IV epithelial OC CR or PR to chemotherapy plus bevacizumab combination	G/sBRCA1/2 and/or genomic instability	III	2020
Rucaparib	NCT01891344	BRCA1/2-mutant, BRCA1/2-wild-type and LOH-high, or BRCA1/2-wildtype and LOH-low recurrent ovarian cancer	Advanced ovarian cancer refractory to ≥ 2 prior lines of treatment	BRCA1/2mutations	II	2016
	NCT01482715	Phase I: advanced stage ovarian cancer Phase II: germline BRCA1/2-mutant ovarian cancer	Ovarian cancer refractory to ≥ 2 prior lines of treatment	BRCA1/2mutations	I/II	2016
	NCT01968213	Recurrent ovarian cancer with PR or CR to most recent line of platinum-based chemotherapy (after ≥ 2 lines chemotherapy	Maintenance therapy for patients with advanced-stage ovarian cancer in CR or PR after platinum-based chemotherapy	Irrespective of BRCA1/2 status	III	2018
Niraparib	NCT01847274	Platinum-sensitive, recurrent ovarian cancer stratified into two subgroups: germline BRCA1/2 mutant and BRCA1/2 wild type (after ≥ 2 lines chemotherapy	Maintenance therapy for patients with advanced stage ovarian cancer who are in CR or PR after platinum-based chemotherapy	–	III	2017

Besides OCs, TNBC is among the most lethal diseases affecting women with few targeted therapies [30]. BC is a heterogeneous disease including more than 20 histologic types, among which TNBC represents the main BC type with BRCA1/2 mutations [31]. Success regarding DDRi in OC treatment has paved the way for clinical trials in BCs. Two PARPi, olaparib [32] and talazoparib [33], are now approved in HER2-negative, BRCA1/2-mutant advanced BCs (Table 2). Although testing of DDRi combination with ICBs is being pursued in a growing number of clinical trials worldwide for a wide range of cancers, currently available data are inconsistent, even in patients with the same cancer type but different molecular characteristics [34, 35]. Here, we primarily discuss how ICB, DDRi, and their combination, may impact the signaling mechanisms and tumor immune microenvironment (TME) in breast and ovarian cancers with HRD, while proposing strategies to optimize therapy. We also discuss both potential biomarkers for patient stratification and determining different toxicities of PARPi as well as distinct levels of PARP trapping [36], and outline opportunities and challenges regarding the promising combination strategies to overcome ICB resistance.

**Table 2:** FDA-approved DDR inhibitors in breast cancers in the past 5 years (2016–2021)

PARP inhibitors	Trial number	Disease setting	FDA approval	Approved mutation status	Study phase	Approved year
Olaparib	NCT02000622	Metastatic, gBRCA1/2-mutant, HER2-negative breast cancer after ≤ 2 prior lines of chemotherapy	Metastatic, HER2-negative breast cancers	BRCA1/2 mutations	III	2018
Talazoparib	NCT01945775	Advanced and/or metastatic HER2-negative breast cancer with germline BRCA1/2 mutation	HER2-negative locally advanced or metastatic breast cancer	Deleterious or suspected deleterious germline *BRCA* mutation	III	2018

Reviewing these concepts and strategies is timely, given the expectation that an increasing number of BC and OC patients will be treated with DDRi, ICB, or their combination. The progress in these areas has been strikingly rapid, with FDA approvals of four PARPi and multiple immunotherapies in breast and ovarian cancers, respectively.

## Mechanisms of ICB resistance in solid tumors

**Fig. 1:**
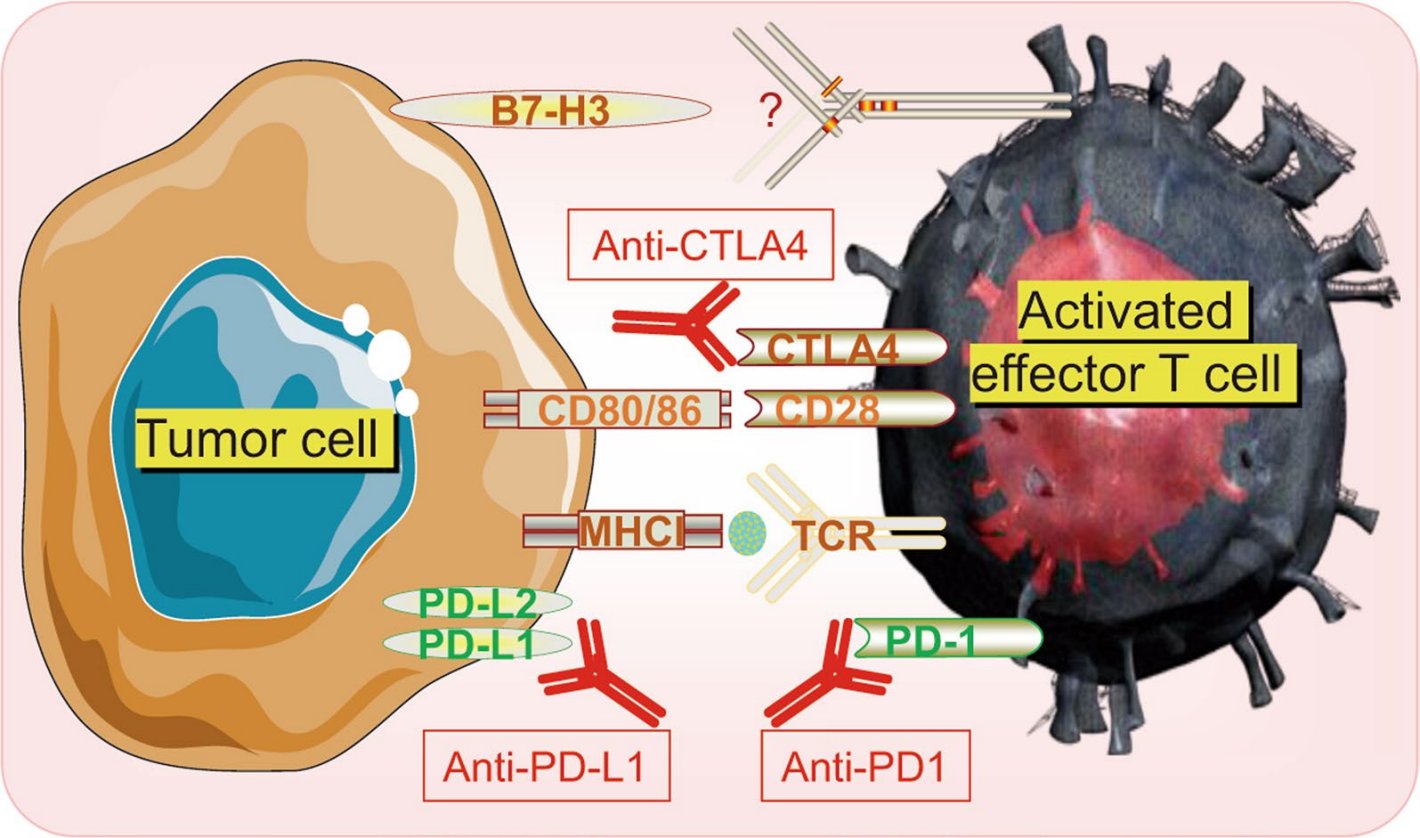
Schematic illustration of tumor cells interacting with activated T cell. Inhibitory immune checkpoints, such as CTLA-4, PD-1, PD-L1 and B7-H3, bind with their partners to blockade T cell activity, while ICBs, such as anti-CTLA-4, anti-PD-1, anti-PD-L1 activate T cell by preventing the interaction cytotoxic T-lymphocyte-associated antigen 4 (CTLA-4); major histocompatibility complex I (MHCI); T cell receptor (TCR); programmed death‐ligand 1 (PD‐L1); programmed death‐1(PD-1); immune checkpoint blockade (ICB)

The major histocompatibility complex (MHC) on the cell surface displays peptides to the T cell receptors (TCR) on antigen-specific T cells to activate anti-tumor T cell responses [37]. Actions of immune checkpoints are mediated through the interactions between the ligands (B7 family members: B7-1, B7-2, PD-L1, B7-H2, B7-H3, and B7-H4) and the receptors (CD28, PD-1, and CTLA-4) [38]. CD28 and CTLA4, the costimulatory receptors, compete for the same ligands, B7-1 (CD80) and B7-2 (CD86) [39]. However, CTLA4 binds these ligands with higher avidity than CD28, allowing CTLA4 to compete with CD28 for ligand and deliver a negative regulatory signal to the T cell [39]. Currently, targeting PD-1, PD-L1, and CTLA-4 with monoclonal antibodies is the mainstay of ICB, with indications for their use in monotherapy or combination in multiple cancers [40]. Importantly, these approaches are markedly different in terms of mechanism of action. For instance, CTLA-4 inhibits T-cell activation, whereas PD-1, another key inhibitor, terminates the effector T-cell responses by interacting with its ligands PD-L1 (B7-H1) and PD-L2 (B7-DC) [41]. Compared with PD-L1, PD-L2 has a two- to six-fold higher affinity for PD-1 [42], while the PD-L1 antibodies blocking the interaction between PD-L1 and PD-1 do not affect PD-L2/PD-1 interaction [43]. PD-L2 also binds repulsive guidance molecule b on macrophages, dendritic cells (DCs), and some epithelial cell types with unknown mechanism [41]. Additionally, B7-H3, one of B7 family members, can co-stimulate proliferation of both CD4+ and CD8+ T cells, but the specific receptor remains unknown [38] (Fig. 1). Despite considerable early successes and tolerable side effects compared to conventional treatments, such as surgery, chemotherapy, radiotherapy (RT), and targeted therapy, the overall response rates of ICB are generally limited [44], being approximately 5‒23% in BCs [45] and 6–15% in OCs [46].

**Fig. 2:**
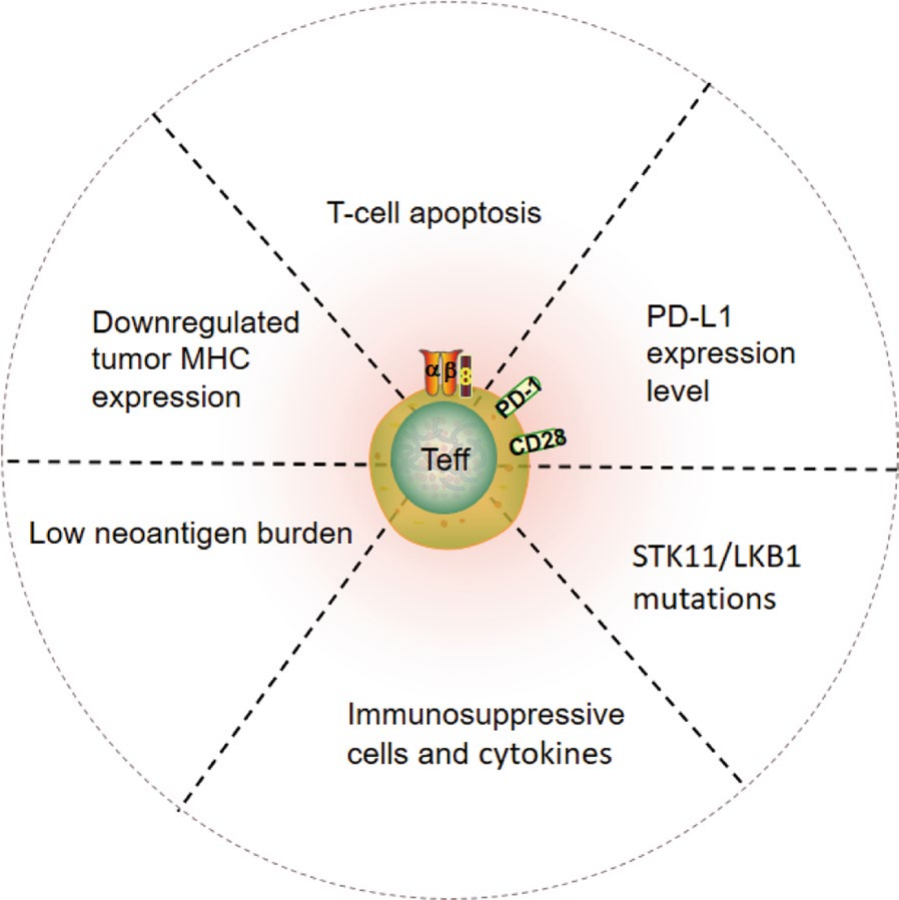
Schematic of resistant mechanisms involving DDR in response to immune checkpoint therapy by influencing *T*_eff_. DNA damage response (DDR); effector T-cells (*T*_eff_); tumor mutation burden (TMB); major histocompatibility complex (MHC)

The widely held view is that activation of effector T-cells (*T*_eff_) is the key beneficial mechanism in human tumors [47]. Thus, the prevailing hypothesis is that the efficacy of ICB is limited to patients with pre-existing antitumor immunity and *T*_eff_ infiltration, while resistance to ICB, which is observed in most cancer patients, is mediated by barriers that impact *T*_eff_ infiltration and/or activity. These barriers are multifactorial and the resistance mechanisms that can be reversed by inhibition of DDR signaling pathways [48–53]. These factors may include low neoantigen expression and downregulation of MHC expression on the cancer cells, imbalance in immune checkpoint expression, increased tumor infiltration by suppressive immune cell populations, tissue hypoxia, tumor metabolic status, and immunosuppressive cytokines. Here, we focus on the resistance mechanisms related to DDR signaling pathways (Fig. 2).

### Low neoantigen burden

Genomic analyses have revealed that mutational processes altering DDR and repair pathways impact responses to ICB, thus influencing clinical outcomes in patients treated with ICB [6, 54]. Non-synonymous somatic alterations presented by tumor cells can be recognized by the immune system as non-self and neoantigens, that is, tumor-associated antigens eliciting T-cell responses [55]. Moreover, TMB serves as a surrogate marker for tumor neoantigen load, representing the number of cancer cell mutations. Meanwhile, elevated TMB was proposed to increase the chances of generating immunogenic neoantigens and the true neoantigen burden, that is, the number of alterations actually targeted by T-cells may have a stronger impact on ICB response than TMB [55]. For example, tumors with high microsatellite instability (MSI-H) accumulate considerable somatic mutations secondary to deficits in DNA MMR. The resultant high mutational burden renders tumors immunogenic and sensitive to PD-1 ICB, while many patients with MMR-proficient (pMMR) tumors fail to respond to PD-1 blockade therapy [56]. For instance, tumors with MMRd show 20/20 (100%) pathological response, while pMMR early stage colon cancers correlate with poor response rates with 4/15 (27%) pathological responses [57]. Despite the weaker impact of homologous recombination (HR) deficiency on TMB relative to MMRd, catastrophic DNA damage driven by DDRi can be favorable to enhance ICB therapy efficiency [49].

However, MMRd-induced mutations are more likely to be predominantly subclonal, triggering highly heterogeneous tumors [58]. Mcgranahan et al. demonstrated that subclonal (branch) neoepitopes is less effective than clonal neoantigens (trunk) in driving tumor clearance with ICB [59]. Moreover, among lung cancer patients receiving ICB treatment, tumors without certain DNA repair mutations exhibit a lower response rate relative to those with such mutational signatures [60, 61]. Collectively, tumors with deficiencies in DDR pathways respond better to ICB due to elevated neoantigen load [48].

### PD-L1 expression level

In addition to TMB, PD-L1 expression is evaluated histologically as another primary biomarker for ICB therapies in cancer patients [62]. PD-L1 expression from both malignant and immune cells can be stimulated by inflammatory cytokines, such as interferons (IFNs) and work concretely to functionally modulate the cytotoxic T-cell lymphocytes (CTLs) in the TME [63]. Moreover, interferon regulatory factor 7 (IRF7) enhances constitutive PD-L1 expression in an IFN-γ independent fashion through directly inducing transcription of PD-L1 [64]. Therefore, comprehensive and dynamic evaluation of both the global PD-L1 level and IFN expression on T-cells and tumor cells, rather than monitoring only surface PD-L1 on tumor cells, should be a more effective approach for predicting responses to ICB. Additionally, DNA damage, amplified by DDRi, can induce PD-L1 expression by activating both IRF1 and IFNs, providing further rational for combinatorial ICB and DDRi therapeutic strategies [50].

### T-cell apoptosis

A vital component of T-cell-mediated antitumor immune responses is the strong tumor-reactive T-cell infiltration into tumor tissues. The International tumor-infiltrating lymphocyte (TIL) Working Group has developed a standardized methodology for BC TILs used in clinical practice [65]. However, even for tumors with a substantial number of TILs prior to treatment, ICB can be rendered ineffective due to the dysfunctional T-cell phenotype [66].

T-cell apoptosis, a mechanism of tumor-induced T-cell dysfunction, can be triggered by binding of Fas, DR3, DR4, DR5, and TNFR1 on T-cells, with their respective ligands, thereby mediating immune evasion [67]. Impairing DNA repair machineries via KML001, a telomere-targeting drug, also reportedly blocks cell proliferation, cytokine production, and promotes apoptosis of T-cells via suppression of telomeric repeat binding factor 2 (TRF2), telomerase, topoisomerase I and II alpha (Top1/2a), and ATM kinase activities [68]. Moreover, KML001 triggers caspase-3-dependent T-cell apoptosis via telomeric DDR, while caspase-3 cleaves PARP during apoptosis [69]. Additionally, accelerating DNA damage by DDRi may serve to reprogram the TME inflammatory milieu [70], driving the recruitment and infiltration of T-cell into the tumor bed [49], thereby remodeling “cold tumors” to “hot tumors”, via activation of the immune response, as well as dysfunctional T-cell phenotypes and subsequent apoptosis.

### Downregulated tumor MHC expression

The loss or downregulation of tumor MHC-I expression constitutes a main tumor escape mechanism from T-cell-mediated immune responses via influencing the degree and composition of the immune cellular infiltration [71]. Conway et al. showed that the loss of MHC-I/II expression contributed to resistance to ICB [62]. However, DNA damage induced by DDRi can enhance radiation-induced tumor cell MHC-I surface expression [51], supporting the use of DDRi for the reversal of ICI resistance by increasing MHC-I expression.

### Role of immunosuppressive cells and cytokines in tumors

Various immune cell subpopulations are identified as pro-tumorigenic due to their contributions toward an immunosuppressive environment, including Tregs, M2-type tumor-associated macrophages (TAMs), plasmacytoid DCs, N2-type neutrophils, and myeloid-derived suppressor cells (MDSCs) that directly or indirectly inhibit CTL responses.

CD4+CD25+Foxp3+ Tregs exert their non-specific immune suppression via modulating either T-cells or antigen presenting cells (APCs) in a cell‐to‐cell contact-dependent manner by producing inhibitory cytokines, including IL-10 and transforming growth factor beta (TGF-β) [72]. In fact, AZD6738, a ATR inhibitor (ATRi), reportedly correlates with Treg infiltration in lung cancer and colorectal cancer murine models [51, 73].

Macrophages are designated TAMs once they have migrated to tumors. M1 macrophages are the effector cells that participate in tumor-cell-killing role via secretion of cytokines (IL-12 among others) [74]. Alternatively, M2 TAMs promote tumor progression within the TME via hampering CD8+ T-cell responses [75]. Of note, SRA737, a CHK1 inhibitor, affects immunosuppressive M2 TAMs as well as MDSC populations via induction of immunomodulatory factors, including type I IFNβ, CCL5, and CXCL10, and exerts a synergistic effect with anti-PD-1/PD-L1 therapy and low doses of gemcitabine [52]. MDSCs are a heterogeneous population of immature myeloid cells that expand in response to soluble factors generated by tumor and stromal cells and disrupt major mechanisms of antitumor immune response [76]. In fact, depletion of MDSCs in a murine BC model, following treatment with ibrutinib, an irreversible inhibitor of Bruton's tyrosine kinase, significantly improved the efficacy of immune-based therapies, including that of anti-PD-L1 therapy [76].

Taken together, immunosuppressive cells may inhibit the functionality of activated lymphocytes and induce CTL exhaustion via immunosuppressive cytokines, while DDRis have the potential to impact immunosuppressive cells.

### Others

Various signaling pathways, including MAPK, JAK-STAT, PI3K-AKT, WNT-β-catenin, and Hippo pathways, highly correlate with tumor formation and evolution, while alterations in genes associated with these pathways impact the response to ICB [62, 77]. For example, *phosphatase* and *tensin homolog* (*PTEN*), a suppressive gene, negatively regulates PI3K/AKT signaling and suppresses tumor development via dephosphorylating PIP3 [78]. Moreover, loss, inactivation, or attenuation of *PTEN* is the most common genomic aberration of the PI3K and interactive pathways in various types of malignancies, with *PTEN* loss of heterozygosity (LOH) reported in hepatocellular (57% of patients), colorectal (48%), gastric (36%), prostate (52%), and endometrial (49%) cancers [79]. *PTEN* loss has also been linked to DSB repair by regulating the DDR protein RAD51, and CHK1, and has been identified as a predictive marker for PARP inhibitors [80]. Meanwhile, the ATM inhibitor KU-60019 was specifically toxic in *PTEN*-deficient cancer cells and tumor xenografts compared to wild-type cells [81].

A clinical trial reported that STK11/LKB1 mutations, the most prevalent genomic driver of PD-1 blockade resistance, are negatively related to therapy in *KRAS*-mutant lung adenocarcinoma [53]. Meanwhile, another study demonstrated that even in the setting of LKB1 loss, *KRAS-*mutant lung cancers remained treatment-refractory and resistant to ICB [82]. Mechanistically, LKB1 loss results in silencing of stimulator of interferon genes (STING) and insensitivity to cytoplasmic double-strand DNA sensing, while promoting immune escape. Additionally, upon cytoplasmic DSB, STING can activate TBK1/IKKε, which subsequently stimulates release of cytokines/chemokines, including IL-6 and CCL5, and ultimately generates an immunosuppressive TME that impairs ICB response [82].

Taken together, increasing evidence reveals that aberrant pathway proteins related to DDR can contribute to ICB resistance in a myriad of cancers.

## The potential effects of DDRi on the immune system

**Fig. 3:**
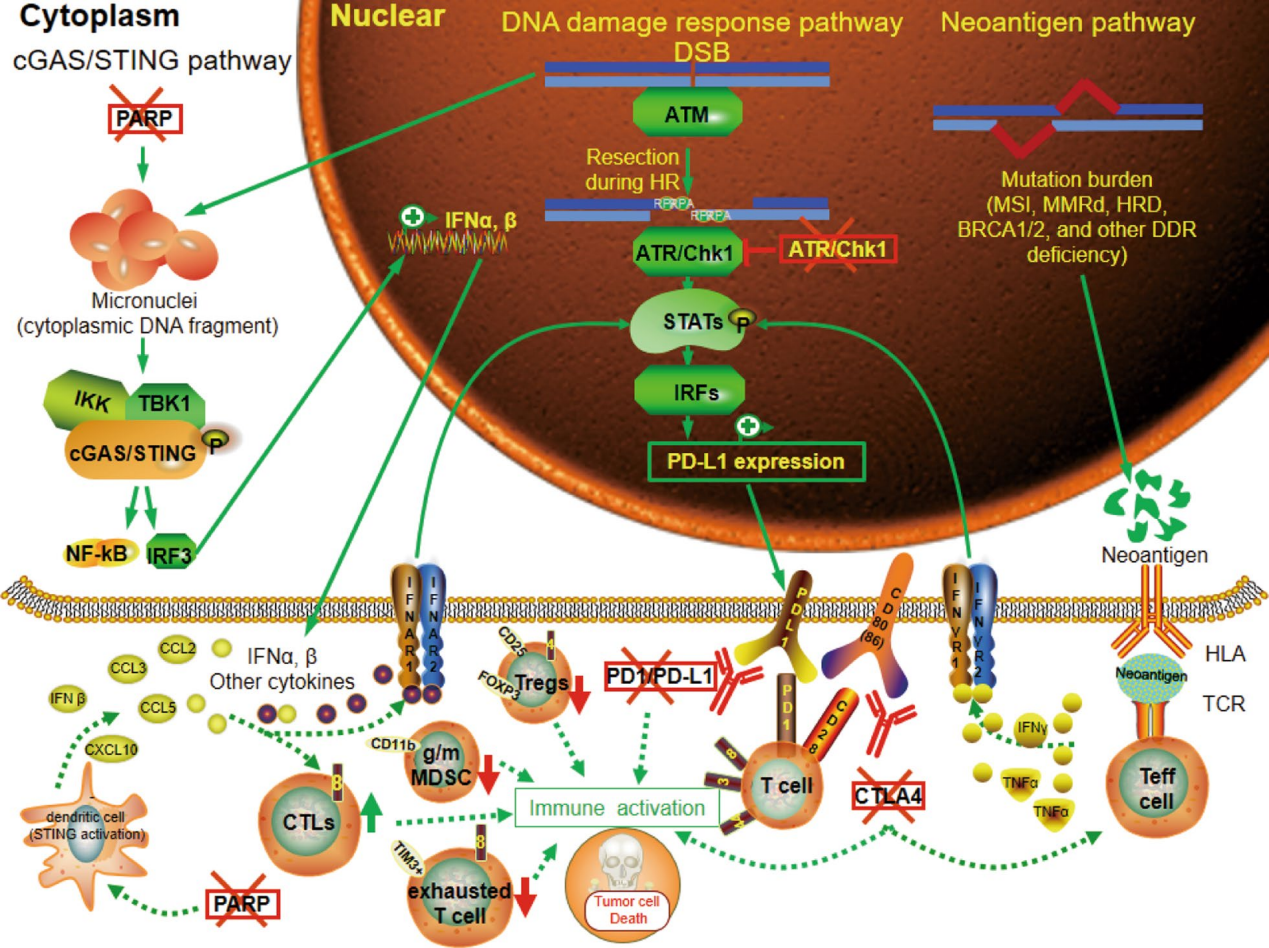
Mechanisms of DDRi and ICB affecting PD‐L1 expression and TME in tumors with DDR deficiency. DNA damage amplified by DDRi activates cGAS/STING, DNA damage response, and neoantigen pathway, inducing PD-L1 expression, pro-inflammatory cytokines release and CTLs infiltration while reducing Tregs and exhausted T cells, which combines with ICB, leading to immune activation and immunogenic cell death. Cyclic GMP‐AMP synthase (cGAS); stimulator of interferon genes (STING); double-strand breaks (DSB); homologous recombination (HR); microsatellite instability (MSI); mismatch repair deficiency (MMRd); homologous recombination deficiency (HRD); breast cancer 1/2 (BRCA1/2); DNA damage response (DDR); T cell receptor (TCR); programmed death‐ligand 1 (PD‐L1); programmed death‐1(PD-1); cytotoxic T-lymphocyte-associated antigen 4 (CTLA-4); cytotoxic CD8+ T cells (CTLs); tumor-necrosis factorα (TNFα); interferon γ(IFN γ); interferon alpha/beta receptor (IFNAR); interferon regulatory factors (IRFs); regulatory T cells (Tregs); immune checkpoint blockade (ICB); granulocytic/monocytic myeloid-derived suppressor cells (g/mMDSCs); poly-ADP-ribose polymerase (PARP); ataxia telangiectasia and Rad3-related protein (ATR); checkpoint kinase 1 (CHK1); effector T-cells (*T*_eff_)

Impressive durable responses to ICI occur in a relatively small fraction of cancer patients, providing an opportunity to test combination strategies that will have more wide-reaching impact. Intrinsic and acquired ICB resistance has directed research toward novel combination treatment strategies aimed at transforming a higher proportion of non-responders into responders in BCs and OCs [83]. Therefore, combination of ICB with other immune-activating strategies, such as DDRi, might be a promising approach to overcome ICB resistance in cancer patients who are PD-L1-negative or PD-L1 positive but acquire resistance to ICB. Considering that DDR proteins maintain genome integrity, amplifying DNA damage using DDR pathway inhibitors may effectively increase the TMB and neoantigen production, alter the inflammatory milieu of the TME, and trigger immunogenic cancer cell death, subsequently activating antitumor immune responses (Fig. 3). Thus, combination of DDRi with ICB, such as anti-PD-1/PD-L1 or anti-CTLA-4 ICB, may initiate antitumor immunity, mediating durable tumor regression (Table 3). Here, we mainly discuss how the efficacy of ICB in the treatment of TNBC and OCs is augmented by DDR inhibition.

**Table 3:** Preclinical DDRi and ICB combination studies with immune read-outs in solid cancer

DDR inhibitors	DDR agent	Immune checkpoint inhibitors	Murine tumor model	Immunological effects	References
PARP inhibitors	Talazoparib (BMN673)	Anti-PD-L1	ID8 (ovarian cancer), CT26 (colorectal cancer)	PARPi increased percentages of CD8+ T cells and PD-L1+ cells; targeting PD-1/PD-L1 pathway potentiates therapeutic efficacy of PARPi	[150]
	Olaparib, talazoparib	Anti-PD-L1	MDA-MB-231, BT549, SUM149 (breast cancer)	PARPis increased PD-L1 expression and decreased tumor-infiltrated cytotoxic CD8+ T-cell population, the addition of anti-PD-L1 restored the cytotoxic CD8+ T-cell population	[97]
	Rucaparib (CO-338)	Anti-PD-1/PD-L1	BRCA1 mutant BrKras (ovarian cancer)	The combination of rucaparib with PD-1 or PD-L1 inhibition improved survival, immune profiling studies are ongoing	[106]
	Olaparib	Anti-PD-1	PBM (Trp53−/−, Brca1−/−, c-Myc) (ovarian cancer)	Olaparib increased PD-L1, Tim-3, and Lag-3, number of intratumoral effector CD4+ and CD8+ T cells, immune cell (CD45+) infiltration, derived a STING-dependent type I IFN signal, IFNγ and TNFα production, reduced MDSCs, the addition of PD-1 blockade overcome limits of PARPi effectiveness and prolonged survival	[102]
	Niraparib	Anti-PD-1	MDA-MB-436 (BRCA1 mutant triple-negative breast cancer), SK6005 (skin tumor), BrKras (BRCA1-deficient ovarian tumor), KLN205 (lung squamous cancer)	Niraparib enhanced the infiltration of CD8+ cells and CD4+ cells and activated interferon pathway, combination with pembrolizumab established immune memory	[107]
	ABT-888 (Veliparib)	Anti-CTLA-4, Anti-PD-1/PD-L1	BRCA1−ID8 (epithelial ovarian cancer)	PARPi in combination with CTLA-4 antibody, but not PD-1/PD-L1 antibody, increased proportion of CD8 cells with an effector/memory phenotype among T cells and enhanced Th1 T-cell response in the peritoneal tumor environment	[109]
ATR inhibitors	BAY1895344	Anti-PD-1/PD-L1	A20 (lymphoma), MC38 and CT26 (CRC)	ATR inhibitors depends on CD8+ T cells to exert the anti-tumor activity	[113]
CHK1 inhibitors	Prexasertib, olaparib	Anti-PD-L1	mTmG (SCLC)	DDRi enhanced PD-L1, CXCL10 and CCL5 mRNA, combination DDRi with anti-PD-L1 enhanced CD3+ total T-cell or CD8+ cytotoxic T-cell, CD44+ memory/effector T-cell, reduced tumor-infiltrating PD-1+/TIM3+ exhausted CD8+ T, CD25+/FOXP3+CD4+ T-regulatory cells, CD62L+ naïve T-cell, and CD4+ helper T-cell infiltration	[151]

*DDR* DNA damage repair, *PARP* poly-ADP-ribose polymerase, *PD-L1* programmed death‐ligand 1, *PD-1* programmed death‐1, *BrKras* BRCA1−/−, P53−/−; myc; Kras-G12D; Akt-myr; *STING* stimulator of interferon genes, *IFN* interferon, *CTLA-4* cytotoxic T-lymphocyte-associated antigen 4, *ATR* ataxia telangiectasia and Rad3-related protein, *CRC* colorectal cancer, *SCLC* small cell lung cancer

### Enhancing tumor antigen release

Inhibition of DDR in sensitive tumor cells, such as those with MMRd, DNA modification, and replication errors, or other alterations of DDR genes including *BRCA2, PRKDC, RAD51C, LIG3, and RAD17*, leads to accumulation of DNA damage, genomic instability, TMB, mutation-associated neoantigens, and ultimately cell death [70, 84, 85]. Mutation-associated neoantigens can activate an adaptive immune response to selectively target cancer cells. An enhanced mutation and neoantigen load may be associated with an improved T-cell response against neoantigens presented by cancer cells, which can be strengthened by ICB [85]. For example, neoantigens presented by MSI-positive tumors augment the release of IFN-γ from TILs, particularly T-cells, thereby elevating PD-L1 levels in tumors and immune cells [86]. IFN-γ stimulates the STAT1/2/3/IRF1 pathway by binding to the IFN-γ receptor, thus triggering PD-L1 expression [50]. Tumors and immune cells with HRD also present enhanced neoantigen burden, TILs, and PD-1/PD-L1 level in BRCA1/2-mutated OCs [87] (Fig. 3).

BCs with germline BRCA1/2 (gBRCA1/2) mutations account for only 5–7% BC cases [88]. By contrast, high-grade TNBC is characterized by a high mutational rate [89]. Along with BRCA mutations, genomic loss of heterozygosity (LOH), large-​scale translocations (LSTs), and telomeric allelic imbalance (TAI) [29], might also be molecular hallmarks of HRD [22]. Although both somatic and gBRCA1/2 aberrations, resulting in high tumor TMB, have been approved by the FDA to function as a companion diagnostic tool for only *BRCA1/2-*mutated OCs [9], the antitumor activity of PARPi in BRCA wild-type tumors has been gaining attention, called “beyond BRCA” efficacy [90].

Substantial mutant neoantigens in MMRd cancers make them sensitive to ICB therapy in 12 different tumor types [91, 92], indicating a pan-tumor biomarker function for ICB efficiency. However, MMRd was only observed in around 1% of BCs, with 1.8% in TNBC [93], indicating that its use should be restricted to high-risk individuals. Low germline MMR gene mutations (2%) with low TMB and inconclusive evidence regarding MMRd were also noted in OCs, while a clinical relevance for immunogenic biological features and MMRd was observed among MSI subset of clear cell OCs [94], indicating that OCs do not have to be regarded as “non-immunogenic” malignancies.

In conclusion, high levels of mutations and neoantigens due to DDR inhibition [95] activate the mutation/neoantigen/IFN-γ pathway, leading to increased PD-L1 expression. To this end, genomic instability in HRD tumors potentially impacts responsiveness to DDR inhibitors when used in conjunction with ICB [96].

### PD-L1 expression

In the TME, the signals from dying or already dead cells transmitted by damage-associated molecular patterns (DAMP) exposed to replicative stress or DNA damage upon DDRi not only promote immune priming, but also induce adaptive upregulation of PD-L1 levels in vitro and in vivo [97]. Mechanistically, PARPis contribute to S-phase–specific DNA damage induced by collapsed replication forks or under-replicated DNA, which subsequently induces accumulation of mitotic chromosomal bridges and micronuclei formation in the G1 phase when cells with DSBs enter mitosis [98, 99]. Cytosolic DAMP is recognized by cyclic GMP‐AMP synthase (cGAS) via generation of the second messenger, cyclic GMP–AMP (cGAMP), which leads to subsequent binding and recruitment of STING in cancers treated with PARPi, such as BCs [100, 101] and BRCA1-deficient OCs [102]. STING can then recruit and activate the cytosolic kinase IKK and tank-binding kinase 1 (TBK1), contributing to the phosphorylation of STING and activation of various transcription factors, including NF-κB and IRF3, to induce the expression of type I IFNs (IFNα and β), as well as other cytokines [103, 104]. Additionally, the nuclear ATM/ATR/CHK1 kinase signaling pathway can modulate PD-L1 expression following DNA damage by phosphorylating STAT1/3 (in the cytoplasm) in cancer cells [50]. When cells enter the S/G2 phase during the progression of HR, ATM, a sensor of DSBs, is immediately activated at the DSB site and the DSBs undergo resection [105]. The generated ssDNA subsequently becomes coated with RPA and activates ATR/CHK1 at the ssDNA gaps [99]. Activated Chk1 is transported from the nucleus, after which it directly phosphorylates STAT1/3 and activates IRF1, which is responsible for the DSB-dependent PD-L1 upregulation [99] (Fig. 3).

This adaptive upregulation of PD-L1 can exert an immunosuppressive effect, likely blocking the PARPi–mediated immune activation [97]. This effect can be overcome by combining PD-1/L1 blockade with PARPi in both BCs [97] and OCs [106]. In fact, these in vitro and in vivo studies revealed that adding DDRi to ICB restores the cytotoxic CD8+ T-cells [97] and establishes immune memory [107], thereby potentiating therapeutic efficacy.

Alternatively, ATR kinase inhibitors (AZD6738 and VE-821) sensitize cancer cells to T-cell killing by downregulating the cell surface expression of PD-L1 in a proteasome-dependent manner to attenuate the PD-L1/PD-1 interaction in MDA-MB-231 BC cells [108]. Meanwhile, in an HPV-driven malignancy model, ATRi and ATRi-RT therapy could drive PD-L1 mRNA expression primarily in CD45+ CD3− cells, with a relatively minor contribution in tumor cells [51]. The cause for PD-L1 upregulation by PARPi and its downregulation by ATRi may be the different mechanisms employed to activate PD-L1, as described above. Moreover, the variable PD-L1 expression upon ATRi may be due to the varying responses to DNA damage and immunogenicity in different tumor models, as well as the distinct time points chosen for analysis. However, the common feature between these two treatment strategies is their mutual ability to potentiate DNA damage, thereby enhancing antitumor immune responses.

A preclinical study in BR5 mouse OCs harboring deficient *BRCA1* revealed that the CTLA-4 antibody, and not PD-1/PD-L1 blockade, together with the PARPi, veliparib, resulted in immune-mediated tumor remission and long-term survival (*p* < 0.0001) by increasing CTLs with an effector/memory phenotype and inducing IFN-γ and TNFα expression by promoting the Th1 effector phenotype among T-cells [109]. One reason for these contradictory findings is the use of distinct models, with differences in their TME. Another explanation for the selective efficacy of CTLA-4 blockade is that the activation of new lymphocyte clones, instead of the reversal of T-cell exhaustion, contributes to immune-mediated antitumor responses in the BRCA1 model compared to PD-1 blockade [110]. This difference may also result from using different PARPi in terms of the catalytic inhibition and PARP trapping potencies, with veliparib displaying a weaker efficacy in HRD and HR-proficient (HRR) cell lines compared with olaparib, talazoparib, or rucaparib [111, 112].

### Reprogramming the TME

CTLs are the key mediators of antitumor immunity [85]. DDRi could have immunosuppressive effects or improve the antitumor response by influencing CTLs [97]. T-cell priming might rely on IFN-β activation via the STING protein complex to generate an antitumor immune response [85]. For example, olaparib elicits an antitumor immune response by inducing both intra-tumoral and peripheral effector CD4+ and CD8+ T-cells. Upon PARP inhibition, APCs, such as DCs, stimulate a STING-dependent type I IFN signal (IFN-β) and CXCL10 secretion, which are partially responsible for the treatment efficacy of PARPi in a co-culture system of BRCA1-deficient OCs [102]. Besides, olaparib promoted CD8+ T-cell recruitment via activating cGAS/STING signaling in tumor cells with paracrine activation of DCs; this was more obvious in HR-deficient than in HRR TNBC cells and in vivo mouse models [100]. An ATR inhibitor, BAY1895344, enhances antitumor efficacy following PD-1/L1 treatment relying on CD8+ T-cells [113]. Notably, the augmented antitumor efficacy is sequence-dependent and has only been achieved post-application of PD-1/PD-L1 blockade, followed by BAY 1895344 treatment (Fig. 3).

PARPis also reportedly decrease the tumor-infiltrating cytotoxic CD8+ T-cell population, while addition of PD-L1 blockade increases the cytotoxic CD8+ T-cell population, thereby re-sensitizing PARPi-treated tumor cells to T-cell killing in TNBC [97]. Mechanistically, PARPis enhance the level of PD-L1 expression on EMT6 tumor cells, a TNBC cell line, by inactivating the GSK3β pathway in vitro and in vivo, when inoculated into a syngeneic mouse model. This upregulation induces a decline in the tumor-infiltrating cytotoxic CD8+ T-cell population, as determined by quantifying the level of IFN-γ. Hence, neoantigen-specific tumor-infiltrating T-cells become subjected to ICB modulations and are highly heterogeneous [114].

DDRi are also capable of transforming chronic, weak DNA damage, to a more robust level of damage by resetting the inflammatory microenvironment of tumors (Fig. 3). ATR inhibition enhances radiation-induced inflammatory IFN response and cytokine gene expression, either in vivo (particularly CCL2, CCL5, and CXCL10) or in vitro (CCL3, CCL5, and CXCL10) [51]. Inflammatory cytokines increase over days, are driven by DSB formation, and modify the TME by recruiting immune cells and are proven to be key to both local and systemic (abscopal) tumor responses [115]. Meanwhile, micronuclei with cGAS accumulation may initiate inflammatory signaling after RT [115].

Cytokine secretion into the extracellular space exerts a bystander effect on neighboring cells, contributing to an immunogenic TME. For instance, BC cells with DDR deficiency have been linked to increased production of CXCL10 and CCL5, compared to DDR-proficient cells, both of which are important for the chemotaxis of peripheral blood mononuclear cells [101]. HRD and/or DDRi might promote immunological vulnerabilities in tumors, while simultaneously inducing immunosuppressive pathways, providing a rationale for combination with ICB therapy.

Taken together, DNA damage enhanced via DDRi yields greater mutational burden, increases neoantigen expression, triggers the release of pro- inflammatory mediators, and leads to greater immune recognition of the tumor, thus increasing the level of inflammatory cytokines and TILs.

## Biological role of DNA damage amplified by DDRi in cancer cells

Mutation of genes involved in the DNA MMR pathway, as well as in other DNA damage repair pathways, is reportedly enriched in patients who exhibit durable clinical benefit from ICBs [9]. Thus, using targeted agents for DDR pathways, including inhibitors of ATR/CHK1 [116], PARP [117], ATM, cyclin-dependent kinase 4/6 (CDK4/6), DNA-PK, WEE1, and aurora kinase B (AURKB) [9] opens new exciting avenues for clinical development of ICB for cancer treatment.

DDR is a complex network of signaling pathways involving DNA damage repair, cell cycle checkpoints, and apoptosis [118] that is key to ensuring overall genomic stability and cell viability, and is responsible for repairing the two primary forms of DNA damage: single-strand breaks (SSB) and DSB.

In response to DSB, the Mre11–Rad50–Nbs1 complex stimulates the ATM-CHK2 pathway, stabilizing p53 via phosphorylation, and causing G1 arrest in normal cells [116]. However, due to frequent inactivation of p53 or retinoblastoma (RB) proteins, most cancer cells exhibit dysregulated G1 checkpoints with insufficient time for DDR to occur prior to DNA replication, causing cells to rely exclusively on intra S and G2/M checkpoints activated by the ATR-CHK1 pathway upon stalled replication forks [99]. Hence, therapeutic inhibition targeting ATR has been shown to increase selective killing of tumor cells [116]. Cancer cells may reportedly depend on the ATR-CHK1 signaling pathway and intact S/G2-M checkpoints for repairing DNA damage [116], and thus, ATR inhibition may result in selective cytotoxic DNA damage and detrimental mitotic catastrophe to tumor cells, while normal cells with a functional G1 checkpoint will be unaffected. This also partially explains the essential nature of ATR and/or CHK1 protein kinase for regulating replication stress and ensuring genome integrity and cell survival [119, 120].

Moreover, WEE1 activates the G2/M cell cycle checkpoint by suppressing CDK1/2, while WEE1 inhibition abrogates the G2 checkpoint, resulting in unscheduled entry into mitosis, elevated replication stress, and subsequent nucleotide starvation and genomic defects [9].

Following SSB, PARP enzymes, key eukaryotic stress sensors, can activate various downstream proteins that participate in SSB repair or base excision repair [9]. Hence, PARP inhibition generates persistent SSB, which ultimately leads to DSB, thereby accelerating replication progression and limiting the ability of the cell to stall DNA replication and repair. This effect is particularly pronounced in cells harboring *BRCA1/2* mutations, in which HR is defective, while in cells without defective HR, accumulated DSBs can be repaired by HR or NHEJ [11]. Notably, the antitumor effects of different PARPi varied due to altered PARP trapping activity, that is, the ability of PARPi to trap PARP-1 on DNA to enhance the stability of PARP-DNA complexes [36].

DNA-PK, another sensor of DNA damage, is also a critical enzyme for DNA repair via NHEJ [9]. Therefore, disrupting PARP, ATM, ATR/CHK1, WEE1, or DNA-PK activity may selectively contribute to accumulation of DNA damage in tumors, sensitize cancer cells to killing, and ultimately induce cell death. Importantly, DNA repair defects are more abundant and specific in malignant tissues than in normal tissues [35, 121]. Moreover, DNA damage proteins and cancer cells have specific properties, including cancer-specific DDR defects and lack of G1 checkpoint control, that may highlight particular vulnerabilities of the cancer [122], supporting PARP, ATR, ATM, CHK1/2, and WEE1 as therapeutic targets.

**Fig. 4:**
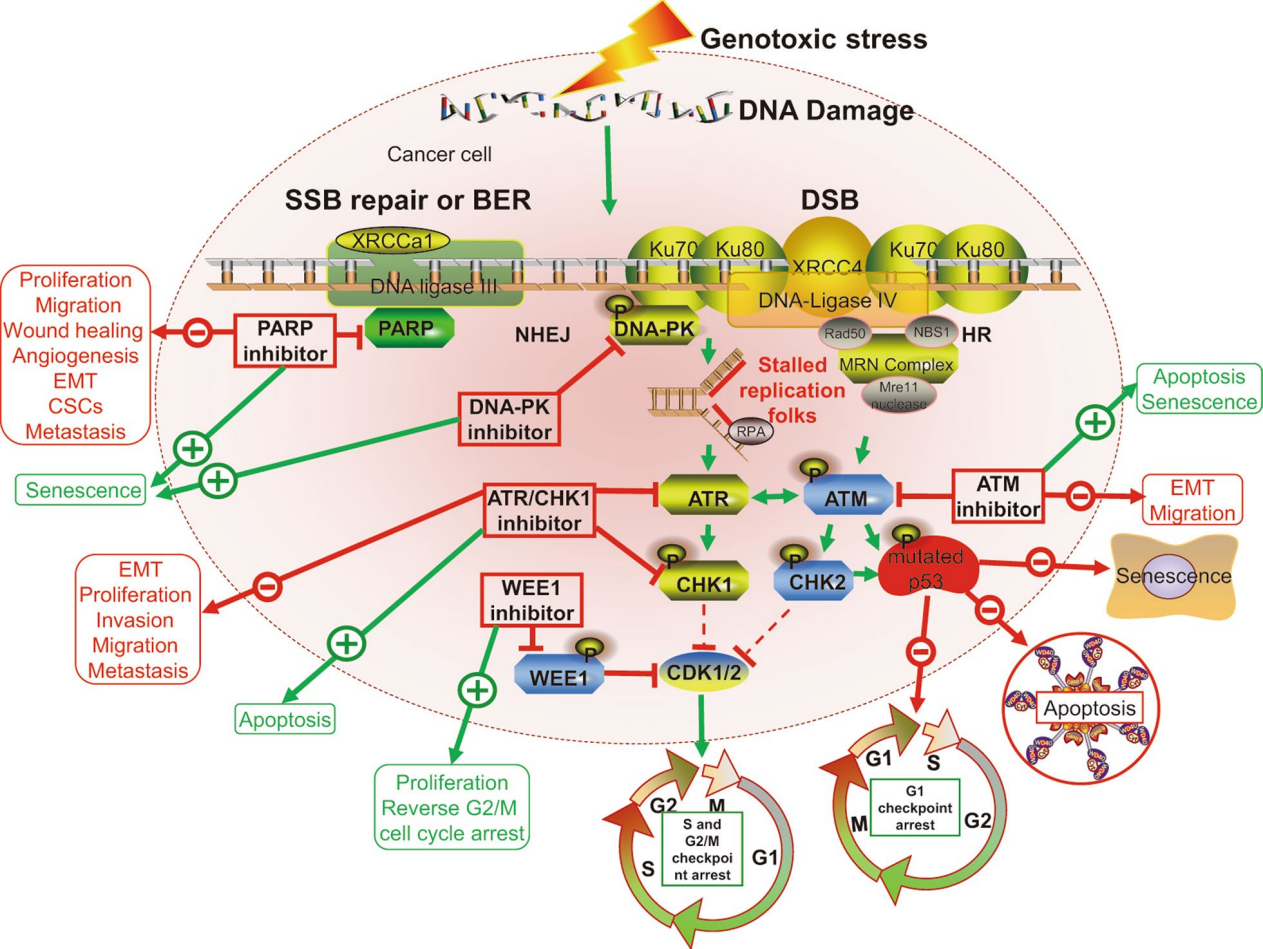
Biological role of targeting DDR protein upon DNA damage in cancer cells. Upon DSB and replication stress, ATM, ATR, and DNA-PKcs are recruited to DNA damage sites, and ATM/CHK2 and ATR/CHK1 pathways are activated. In normal cells, ATM activates p53 by phosphorylation, leading to G1-phase arrest, senescence and apoptosis. However, in tumor cells, p53 is inactivated frequently, disrupting the G1-S cell cycle checkpoint, and making the cells dependent on G2-M cell cycle checkpoint for arrest upon DNA damage. The phosphorylation of WEE1 abolishes the activation of CDK1/2, inducing G2/M cell cycle arrest. PARP enzymes are primary proteins involved in SSB repair or base-excision repair (BER). Single-strand break (SSB); base excision repair (BER); double-strand breaks (DSB); non‐homologous end joining (NHEJ); homologous recombination (HR); ataxia telangiectasia mutated protein (ATM); poly-ADP-ribose polymerase (PARP); ataxia telangiectasia and Rad3-related protein (ATR); DNA-dependent protein kinase (DNA-PK); checkpoint kinase 1/2 (CHK1/2); cancer stem cells (CSCs); epithelial–mesenchymal transition (EMT)

Inhibition of specific DDR proteins, which normally halt the cell cycle in DNA damage repair pathways, may play various roles in different physiological and/or pathological events, particularly during neoplasia. For example, PARPi inhibit metastatic action and tumor recurrence via modulating the hypoxic response and suppressing proliferation, epithelial–mesenchymal transition (EMT), angiogenesis, and cancer stem like cells across different tumor settings [123]. Moreover, inhibition of the ATR/CHK1 signaling pathway blocks EMT, cell proliferation, invasion, migration, tumorigenicity, and lymph node metastasis, while inducing apoptosis in cervical cancer [124]. Certain DNA damage sensors, such as ATM, ATR, FOXO3a and p53, function as critical regulators of autophagy and are critical to the maintenance of cell cycle arrest and DNA repair activities in histiocytic lymphoma cells [125]. Conversely, in most malignancies, autophagy can support DNA synthesis, thereby conferring survival to tumor cells under replication stress [126], whereas autophagy inhibition with niraparib treatment, a PARPi, accelerates DNA damage and cell death in laryngeal squamous cell carcinoma [127]. Moreover, DNA damage-induced senescence physiologically and pathologically correlates with aging and age-related diseases in vivo [128]. For instance, PARPi can induce senescence in BC and high-grade serous epithelial OC cells [129]. Additionally, AURKB inhibitors trigger senescence in non-small cell lung cancer cells with acquired mutations via the ATM/Chk2 DDR [130]. Nevertheless, in patients with breast tumors, low expression of XRCC1, ATM, and BRCA1 correlates with high proliferation indexes, higher tumor grade, and the presence of dedifferentiated cells [131]. Taken together, DDRi suppress adhesion, proliferation, migration, invasion, EMT, formation of autophagosome, and angiogenesis [132], while stimulating apoptosis and senescence by directly targeting DNA damage repair processes and augmenting DNA damage in cancer cells. Moreover, DNA damage, while tumor-suppressive, promotes pro-carcinogenic effects mediated by the TME (Fig. 4).

## Clinical translation of DDRi plus ICB as a strategy to overcome immunotherapy resistance

Based on these promising data on the combination of DDRi with ICB therapy in preclinical and translational settings, multiple clinical trials are currently ongoing (Additional file 1: Table S1), with results for certain DDRi/ICB combinations having already been reported, including olaparib/durvalumab [133–135], niraparib/pembrolizumab [136], pembrolizumab/olaparib [137], BGB-A317/BGB-290 [138], and AZD6738/durvalumab [139].

The first clinical trial reporting durvalumab plus olaparib demonstrated that the combination therapy had no overlapping toxicity, and the efficiency was established in two patients with a partial response (PR) and eight with stable disease (SD), yielding an ORR of 14% in 35 patients and a disease control rate of 83% in 12 patients harboring advanced-stage BCs [134]. Among them, only a small population achieved a durable response, with a clinical benefit rate (PR + SD ⩾ 6 months) of 34%. The TOPACIO/KEYNOTE-162 trial showed that the combination of niraparib with pembrolizumab exhibited a good tolerability profile, with the ORR (47% vs. 11%) and progression-free survival (PFS) (8.3 months vs. 2.1 months) being higher in BRCA1/2 mutant TNBC patients than in non-mutant ones [140]. Likewise, the MEDIOLA trial showed that in one of the four cohorts (NCT02734004), olaparib plus durvalumab achieved an impressive DCR of 80% and an ORR (all PRs) of 52% for patients with advanced-stage *gBRCA1/2*-mutant BCs, with no obvious overlapping toxicities [141].

In the MEDIOLA trial of a gBRCA1/2-mutated platinum-sensitive relapsed OC cohort, combination of durvalumab with olaparib also exhibited no overlapping toxicity, with ORR of 71.9%, median PFS of 11.1 months, and 28-week DCR of 65.9% [142]. Meanwhile, in the TOPACIO/KEYNOTE-162 trial of an advanced platinum-resistant OC cohort, niraparib plus pembrolizumab achieved a DCR of 68% in the general study population, similar to the 73% DCR in patients with *BRCA1/2* mutations [140], indicating that PARPi plus ICB exhibits antitumor activity, regardless of HR or BRCA status. The combination in both trials was safe with the most common grade 3 or greater AEs being anemia (21% TOPACIO; 9% MEDIOLA) [5]. Notably, a phase I/II trial (NCT02953457) is testing dual blockade of the immune checkpoint using anti-PA-L1 and anti-CTLA4 (durvalumab and tremelimumab) plus PARPi (olaparib) in recurrent or refractory BRCA1/BRAC2-mutated epithelial OCs.

Combination of ICB and DDR inhibitors with other agents, including RT, chemotherapy, anti-angiogenic or epigenetic drugs, is emerging in preclinical and clinical studies based on the good tolerability of the DDRi/ICB combination. RT can kill cancer cells, while simultaneously inducing the release of pro-inflammatory mediators, increasing infiltration of immune cells, and promoting the expression of neoantigens [84]. Meanwhile, DDRi can further enhance the immunogenic effects of RT through augmenting CTL infiltration into the tumor bed, as well as the expression and secretion of chemokines [109], while also promoting PD-1/PD-L1 expression. Therefore, adding ICB to this combination could counterbalance such immunosuppressive effects [143, 144], providing a preclinical rationale for triple combination therapy to improve treatment efficiency of PD-axis ICB in clinical trials. A phase II study (NCT04690855) combining talazoparib (PARPi), RT, and atezolizumab (anti-PD-L1) is currently recruiting gBRCA 1/2 negative patients with PD-L1+ metastatic TNBC. Additionally, more detailed studies on the clinical combinations of RT, DDRi, and ICB are warranted to address concerns regarding subclonal neoantigen generation [84].

Addition of epigenetic drugs to PARPi/anti-PD1/PD-L1 is a latently synergistic strategy for potentiating immunogenic cell death and overcoming resistance due to the additional suppression of cancer stem cells in TNBC [145]. The I-SPY2 trial reported a significantly enhanced pathologic complete response when durvalumab and olaparib were combined with paclitaxel in comparison with chemotherapy alone among women with stage II/III high-risk, HER2-negative BC; improvement was observed in both the HR+ and TNBC subsets [146]. Moreover, clinical trials of evaluating addition of bevacizumab (anti-VEGF humanized monoclonal antibody) to nivolumab plus rucaparib (NCT02873962) or to olaparib plus durvalumab (NCT02734004), niraparib plus dostarlimab (TSR-042, anti-PD1) (NCT03574779 and NCT03806049) are recruiting patients with relapsed or advanced OCs.

Other clinical trials are currently ongoing to investigate the combination of ICB with DDRis, such as durvalumab plus olaparib, AZD6738, or AZD1775, tremelimumab plus olaparib, pembrolizumab plus olaparib, or niraparib, nivolumab plus niraparib, or veliparib, atezolizumab plus olaparib, niraparib, ipilimumab plus niraparib, and BGB-A317 plus BGB-290. Taken together, the effects of these combinations are being evaluated and results are eagerly awaited.

## Biomarkers for predicting clinical benefits of combination DDRi and ICB

Biomarkers for BC and OC treatment response to DDRi plus ICB are limited. In HER2-negative stage II/III BC patients treated with triple combination (durvalumab, olaparib, and chemotherapy) compared with chemotherapy alone, a higher expression of immune gene signatures and PD-L1 mRNA expression was associated with higher pathologic complete response rates [146]. Meanwhile, mutational signature 3, reflecting HRD, and positive immune score, serving as a substitute of interferon-primed CD8-exhausted effector T-cells detected by targeted gene panel sequencing, function as a positive predictive or prognostic marker for platinum-resistant OCs when combining niraparib with pembrolizumab [147]. Further, enhanced systemic IFNγ linked to higher PFS (HR 0.37, *p* = 0.023) and high post-treatment levels of VEGF3R3 correlated with worse PFS in epithelial OC patients treated with olaparib plus durvalumab [10]. In other solid cancer types, there are some predictive and prognostic markers overlapping with biomarkers for ICB and DDRi monotherapy (Table 4).

**Table 4:** Biomarkers that predict response to DDR-targeted therapies in combination with ICB

Factor	Agents	Incidence (%)	Validated in clinical trial?	Association with favorable clinical outcome	Predictive versus prognostic	Cancer type	Tissue type for biomarker assessment	Possible assay type for biomarker assessment
Mutational signature 3 reflecting HRD, IS	Niraparib, pembrolizumab	51%	I/II (NCT02657889)	Positive	Prognostic, predictive, or both	Ovarian cancer	Tumor tissue	Targeted gene panel sequencing
Pre-existing CD8+ T-cell infiltrates	Durvalumab, olaparib	NR	II (NCT02484404)	Positive	Predictive	Relapsed SCLC	Tumor tissue	Immunohistochemistry
MDSCs (≤ the median)	Durvalumab, olaparib	NR	I/II (NCT02484404)	Negative	Prognostic	MCRPC	Blood	Multiparametric flow cytometry
CTC	Durvalumab, olaparib	NR	I/II (NCT02484404)	Positive	Prognostic	MCRPC	Blood	Multiparametric flow cytometry
CD83 expression on CD141+ mDCs, > median percentage of Ki67 + PD-1+ cells among total CD8+/CD4+ T cells, > median percentage of Ki67+ HLA-DR CD8+ and CD4+ T cells	Durvalumab, olaparib	NR	I/II (NCT02484404)	Positive	Prognostic	mCRPC	Blood	Multiparametric flow cytometry

As 15% of unselected TNBC [148] and 17% of high-grade serous OC [90] patients harbor gBRCA mutations, BRCA1/2 alterations resulting in HRD can function as a promising biomarker for DDRi use in both OCs and BCs [149]. However, the use of these genomic aberrations as biomarkers to identify patients who are more likely to benefit from the combination therapy will require extensive validation in large and well-designed clinical trials. Ideally, future studies should integrate the complexity of the biology of both the tumor and its TME (such as PD-L1 level, CD8 T-cell infiltration, and other immune infiltrates) or gene expression profiling of genomic alterations using NGS platforms to establish a deeper understanding of the DNA damage and immune-related biomarker groups, and thus help to precisely guide the clinical development of this new strategy.

## Conclusions and future perspectives

DDRi might generate immunological vulnerabilities in tumors, while concurrently stimulating immunosuppressive signaling, such as PD-1/PD-L1 signaling. Therefore, the combination of ICB with DDRi might overcome ICB resistance, inducing robust antitumor immune responses and immunogenic cell death of cancer cells, resulting in a potential cure, especially in cancers with high incidence of HRD, such as BRCA-mutant BCs and OCs. Moreover, DDRi plus ICBs demonstrated enhanced treatment efficiency in BC and OC patients compared with monotherapy in both first-line and recurrent settings, which primarily depended on early endpoints, such as ORR, making it suitable for patients with limited responsiveness rather than those with high response rate. Therefore, it might be more reasonable to assess clinical benefit in terms of long-term benefits, such as duration of response or OS.

Clinical trials exploring the dual combination in the neoadjuvant setting of BCs have already begun. A phase II trial (NCT04584255) is trying to investigate the synergistic efficacy of niraparib plus dostarlimab in BRCA-mutated BCs and TNBC. However, more effective therapies are still required for BRCA-negative and PDL-L1 negative BCs, as well as non-TNBC subtypes and in the neoadjuvant setting.

In OC patients with both repair-deficient and -proficient status, ongoing phase III studies are trying to explore rucaparib plus nivolumab (ATHENA, NCT03522246), atezolizumab plus niraparib (ANITA, NCT03598270), avelumab plus talazoparib (JAVELIN, NCT03642132), and niraparib plus dostarlimab (anti-PD1) (NCT03602859) in both the frontline treatment and the maintenance setting following platinum-based chemotherapy. These trials may further help answer the question of whether the combinational treatment is confined to HR-deficient or can be extended to the HRR phenotype.

Overall, the combination of DDRi with ICB is promising due to their distinct, mostly non-overlapping toxicities, particularly in BC and OC patients with HRD. However, greater clarity is needed via basic and translational studies to elucidate the mechanism of action of both DDRi and ICB as monotherapies, and in combination. Besides evaluating safety and efficacy in clinical research, validating predictive biomarkers to identify sensitive patients should become a priority.

## Supplementary Information

**Additional file 1. Table S1**: Summary of Clinical Studies Combining Inhibitors Targeting DDR and ICB in Solid Cancer (ClinicalTrials.gov).

## Data Availability

Not applicable.

## Abbreviations

ICB: Immune checkpoint blockade
PD-1: Programmed death receptor 1
PD-L1: Programmed death-ligand 1
CTLA-4: Cytotoxic T-lymphocyte antigen 4
ORRs: Objective response rates
BC: Breast cancers
OC: Ovarian cancers
DDR: DNA damage response
DDRi: DNA damaging response inhibitors
TMB: Tumor mutational burden
PARP: Poly-ADP-ribose polymerase
MMR: DNA mismatch repair
MMRd: MMR deficiency
HRD: Homologous recombination deficiency
BRCA1/2: Breast cancer 1/2
Indel: Insertion and deletion
DSB: DNA double-strand breaks
TNBC: Triple-negative breast cancer
PARPi: PARP inhibitors
CHK1: Checkpoint kinase 1
HRR: HR-proficient
ATM: Ataxia telangiectasia-mutated
ATR: Ataxia telangiectasia and Rad3-related protein
DNA-PK: DNA-dependent protein kinase
FDA: Food and Drug Administration
TME: Tumor immune microenvironment
MHC: Major histocompatibility complex
DCs: Dendritic cells
RT: Radiotherapy
*T*_eff_: Effector T-cells
MSI: Microsatellite instability
pMMR: MMR-proficient
TIL: Tumor-infiltrating lymphocyte
IFNs: Interferons
CTLs: Cytotoxic T-cell lymphocytes
TIL: Tumor-infiltrating lymphocyte
TAMs: Tumor-associated macrophages
MDSCs: Myeloid-derived suppressor cells
APCs: Antigen presenting cells
ATRi: ATR inhibitor
gBRCA1/2: Germline BRCA1/2
STING: Stimulator of interferon genes
cGAS: Cyclic GMP‐AMP synthase
SSB: Single-strand breaks
EMT: Epithelial–mesenchymal transition
SD: Stable disease
