# Supplementary material for: The developing landscape of combinatorial therapies of immune checkpoint blockade with DNA damage repair inhibitors for the treatment of breast and ovarian cancers
Source: J Hematol Oncol. 2021 Dec 20;14:206. doi: 10.1186/s13045-021-01218-8 (PMC8686226; doi:10.1186/s13045-021-01218-8)
Supplement: Supplementary file 1 — Additional file 1. Table S1: Summary of Clinical Studies Combining Inhibitors Targeting DDR and ICB in Solid Cancer (ClinicalTrials.gov). [file 13045_2021_1218_MOESM1_ESM.docx]

**Table S1** Summary of Clinical Studies Combining Inhibitors Targeting DDR and ICB in Solid Cancer (ClinicalTrials.gov)

| **ICB** | **Tumour group** | **Target**  **Population** | **DDR inhibitors** | **Phase** | **Arms** | **Estimated enrollment** | **Trial status** | **Clinical trial.gov reference** |
| --- | --- | --- | --- | --- | --- | --- | --- | --- |
| Durvalumab (MEDI4736) | Bladder | Neoadjuvanttreatments priorto surgery | Olapar(AZD2281) | II | Durvalumab + olaparib | 29 | Completed | NCT03534492 |
|  | Head and neck | Preoperative | Olaparib | II | Durvalumab + olaparib, olaparib + cisplatin, olaparib, no treatment | 41 | Completed | NCT02882308 |
|  | Breast | Triple Negative Breast | Olaparib | II | Olaparib, olaparib + durvalumab | 60 | Recruiting | NCT03167619 |
|  | Breast | neoadjuvant, triple negative or low ER+ breast cancer | Olaparib | I/II | Olaparib + durvalumab | 25 | Recruiting | NCT03594396 |
|  | Solid | Advanced or metastatic solid tumor malignancies. | Olaparib | I | AZD5363 + olaparib + durvalumab | 40 | Recruiting | NCT03772561 |
|  | Breast | Metastatic triple negative breast cancer | Olaparib | II | Olaparib + durvalumab | 28 | Recruiting | NCT03801369 |
|  | Solid | Advanced and unresectable malignant solid neoplasm | Olaparib | I | Copanlisib + olaparib + durvalumab | 102 | Recruiting | NCT03842228 |
|  | Gastric | Advanced gastric cancer,  2nd or higher line | Olaparib | II | Durvalumab + olaparib + paclitaxel | 40 | Recruiting | NCT03579784 |
|  | Prostate | Castration sensitive biochemically recurrent non-Metastatic prostate cancer | Olaparib | II | Olaparib + durvalumab | 32 | Recruiting | NCT03810105 |
|  | Ovarian, recurrent fallopian tube, peritoneal | BRCA1 or BRCA2 mutation, 2nd line | Olaparib | II | Olaparib + tremelimumab + durvalumab | 36 | Recruiting | NCT02953457 |
|  | Ovarian, breast, SCLC, gastric | Advanced | Olaparib | I/II | Bevacizumab + olaparib + durvalumab | 427 | Active, not recruiting | NCT02734004 |
|  | Ovarian, fallopian tube, or peritoneal | Recurrent, platinum-sensitive | Niraparib | III | Bevacizumab + niraparib + dostarlimab |  | Not yet recruiting | NCT03806049 |
|  | Endometrial, uterine | 2nd line (one prior platinum-based chemotherapy) | Olaparib | II | Olaparib + durvalumab | 55 | Recruiting | NCT03951415 |
|  | SCLC | Prior lines platinum-positive | Olaparib | I | Radiotherapy followed by durvalumab, radiotherapy followed by durvalumab + tremelimumab, radiotherapy followed by durvalumab + olaparib | 54 | Recruiting | NCT03923270 |
|  | Breast | 2nd line +, ER-positive and HER2-negative metastatic or locally advanced | Olaparib | II | Olaparib + durvalumab + fulvestrant | 158 | Recruiting | NCT04053322 |
|  | Ovarian, breast, SCLC, gastric | Relapsed SCLC, gBRCAm metastatic HER2-negative breast cancer, gBRCAm platinum-sensitive relapsed ovarian cancer, gastric cancer | Olaparib | I/II | MEDI4736 + olaparib ± bevacizumab | 427 | Recruiting | NCT02734004 |
|  | Ovarian | Newly diagnosed advanced ovarian cancer | Olaparib | III | Carboplatin + paclitaxel + bevacizumab followed by maintenance bevacizumab, carboplatin + paclitaxel + bevacizumab + durvalumab followed by maintenance bevacizumab + durvalumab, carboplatin + paclitaxel + bevacizumab (or optional) + durvalumab followed by maintenance bevacizumab (or optional) + durvalumab + olaparib, | 1056 | Recruiting | NCT03737643 |
|  | Ovarian,  breast,  NSCLC,  Prostate | Platinum resistant or refractory-2nd line+,  persistent or recurrent,  2nd line+,  2nd line+ | Olaparib | I/II | MEDI4736 + cediranib, MEDI4736 + olaparib, MEDI4736 + olaparib + cediranib | 384 | Recruiting | NCT02484404 |
|  | Solid | Advanced solid cancers | Olaparib | I/II | Ceralasertib + carboplatin, ceralasertib + olaparib, ceralasertib + durvalumab | 322 | Recruiting | NCT02264678 |
|  | Breast | Neoadjuvant chemotherapy resistant residual triple negative breast cancer, post-surgical adjuvant | Olaparib | II | AZD6738, olaparib, durvalumab monotherapy | 81 | Recruiting | NCT03740893 |
|  | Ovarian | Platinum-resistant recurrent ovarian cancer | Olaparib | II | Olaparib+cediranib, durvalumab + olaparib, durvalumab + chemotherapy, durvalumab + tremelimumab + chemotherapy, durvalumab + tremelimumab + paclitaxel | 86 | Recruiting | NCT03699449 |
|  | NSCLC | Metastatic, 2nd line + | Olaparib, AZD6738 | II | Durvalumab + olaparib, durvalumab + AZD9150, durvalumab + AZD6738, durvalumab + vistusertib, durvalumab + oleclumab, durvalumab + trastuzumab deruxtecan, durvalumab + cediranib | 320 | Recruiting | NCT03334617 |
|  | Breast | Metastatic triple negative breast cancer, | Olaparib | I | Olaparib + durvalumab | 3 | Active, not recruiting | NCT03544125 |
|  | Urinary bladder | First-Line, platinum-Ineligible patients with unresectable stage IV urothelial cancer | Olaparib | II | Durvalumab + olaparib | 154 | Active, not recruiting | NCT03459846 |
|  | NSCLC | Stage IV NSCLC with activating EGFR mutations and ALK fusions, maintenance Therapy | Olaparib | II | Durvalumab + olaparib vs durvalumab | 401 | Active, not recruiting | NCT03775486 |
|  | Ovarian | Relapse, 2nd line+ | Olaparib | II | Bevacizumab + olaparib + durvalumab | 74 | Active, not recruiting | NCT04015739 |
|  | Solid | Advanced solid tumors | Olaparib | II | Olaparib + durvalumab, cediranib + durvalumab | 90 | Active, not recruiting | NCT03851614 |
|  | Bladder | Metastatic, 2nd/3rd line | Olaparib | I | AZD4547, durvalumab, durvalumab + AZD4547, durvalumab + olaparib, durvalumab + vistusertib, durvaluamb + AZD9150, durvaluamb + selumetinib | 156 | Active, not recruiting | NCT02546661 |
|  | SCLC | Platinum refractory extensive-stage SCLC, 2nd line + | Olaparib, AZD6738,  AZD1775 | II | Durvalumab + tremelimumab, AZD1775 + carboplatin, AZD6738 + olaparib | 72 | Active, not recruiting | NCT02937818 |
|  | Prostate | Biochemically recurrent, 2nd line + | Olaparib | II | Durvalumab + olaparib | 30 | Not yet recruiting | NCT04336943 |
|  | Glioma, cholangiocarcinoma, solid | Isocitrate dehydrogenase or mutated | Olaparib | II | Olaparib + durvalumab | 78 | Not yet recruiting | NCT03991832 |
|  | Endometrial | Maintenance | Olaparib | III | Platinum-based chemotherapy + durvalumab followed by maintenance durvalumab, platinum-based chemotherapy + durvalumab followed by maintenance durvalumab + olaparib | 699 | Not yet recruiting | NCT04269200 |
|  | Solid | Metastatic, 2nd line + | Olaparib | II | Olaparib + immunotherapy (durvalumab + Tremelimumab) followed by maintenance durvalumab | 270 | Not yet recruiting | NCT04169841 |
|  | Bile Duct | 2nd line | AZD6738, Olaparib | II | AZD6738 + durvalumab, AZD6738 + olaparib | 74 | Not yet recruiting | NCT04298021 |
|  | Renal | Post-surgery | Olaparib | II | Cediranib, olaparib, olaparib + cediranib, durvalumab, olaparib + durvalumab | 60 | Not yet recruiting | NCT03741426 |
|  | Gastric | Metastatic, 3rd line | AZD6738 | II | AZD6738 + durvalumab | 60 | Recruiting | NCT03780608 |
|  | NSCLC | 3rd or 4th line | AZD6738 | II | Durvalumab + monalizumab, durvalumab + MEDI9447, durvalumab + AZD6738, docetaxel | 120 | Recruiting | NCT03833440 |
|  | Bile Duct | Advanced, 3rd line+ | AZD6738 | II | AZD6738 + durvalumab | 26 | Not yet recruiting | NCT04298008 |
|  | Epithelial ovarian, fallopian tube or primary peritoneal | 2nd line | Olaparib | I/II | Tremelimumab, tremelimumab + olaparib | 68 | Active, not recruiting | NCT02485990 |
| Tremelimumab | Ovarian, fallopian tube, peritoneal | Platinum-sensitive recurrent, 2nd line | Olaparib | II | Olaparib, olaparib + tremelimumab | 170 | Recruiting | NCT04034927 |
|  | Ovarian, fallopian tube, peritoneal | Recurrent, BRCA1 or BRCA2 mutation, 2nd line+ | Olaparib | I/II | Olaparib + tremelimumab | 50 | Recruiting | NCT02571725 |
|  | SCLC | Extensive stage, 2nd line | Olaparib | I | Radiotherapy + durvalumab, radiotherapy + durvalumab + tremelimumab, radiotherapy + durvalumab + olaparib | 54 | Recruiting | NCT03923270 |
|  | Solid | Advanced, 2nd line | Olaparib | II | Nilotinib, everolimus, sorafenib, lapatinib, pazopanib, olaparib, durvalumab + tremelimumab | 560 | Recruiting | NCT02029001 |
|  | Solid | Metastatic and/or unresectable | Olaparib | II | Olaparib + pembrolizumab | 300 | Recruiting | NCT04123366 |
| Pembrolizumab (MK-3475) | Breast | Recurrent, 2nd line or metastatic | Olaparib | II/III | Pembrolizumab + carboplatin + gemcitabine, pembrolizumab + olaparib | 932 | Recruiting | NCT04191135 |
|  | Cholangiocarcinoma | Advanced, 2nd line | Olaparib | II | Pembrolizumab + olaparib | 29 | Recruiting | NCT04306367 |
|  | NSCLC | Squamous, 1st line | Olaparib | III | Pembrolizumab + carboplatin + taxane + maintenance pembrolizumab + olaparib, pembrolizumab + carboplatin + taxane + maintenance pembrolizumab | 735 | Recruiting | NCT03976362 |
|  | NSCLC | Metastatic nonsquamous, 1st line | Olaparib | III | Pembrolizumab + pemetrexed + platinum + maintenance pembrolizumab + olaparib, pembrolizumab + pemetrexed + platinum + maintenance pembrolizumab + pemetrexed | 792 | Recruiting | NCT03976323 |
|  | Prostate | Metastatic castration-resistant, 2nd line + | Olaparib | III | Pembrolizumab + olaparib, abiraterone + prednisone or enzalutamide | 780 | Recruiting | NCT03834519 |
|  | Ovarian, fallopian tube, peritonea | Maintenance | Olaparib | III | Carboplatin/paclitaxel + pembrolizumab + maintenance olaparib | 1086 | Recruiting | NCT03740165 |
|  | Prostate | Metastatic castration-Resistant, 2nd line + | Olaparib | I | Pembrolizumab + olaparib, pembrolizumab + docetaxel + prednisone, pembrolizumab + enzalutamide, pembrolizumab + abiraterone + prednisone | 400 | Recruiting | NCT02861573 |
|  | Gastric | Advanced, 2nd line | Olaparib | II | Paclitaxel + olaparib + pembrolizumab. | 36 | Not yet recruiting | NCT04209686 |
|  | Breast, ovarian | Metastatic triple-negative breast cancer or recurrent ovarian cancer, 2nd line+ | Niraparib | I/II | Niraparib + pembrolizumab | 122 | Active, not recruiting | NCT02657889 |
|  | Pancreatic | Advanced or metastatic, 1st line | Niraparib | I/II | Niraparib + nivolumab, niraparib + ipilimumab | 84 | Recruiting | NCT03404960 |
| Nivolumab | NSCLC | Metastatic | Veliparib | I | Veliparib + nivolumab + carboplatin/paclitaxel or carboplatin/pemetrexed, Veliparib + carboplatin/paclitaxel or carboplatin/pemetrexed | 25 | Completed | NCT02944396 |
|  | Solid | Refractory to standard therapy, advanced | Veliparib | I | Veliparib + nivolumab | 50 | Active, not recruiting | NCT03061188 |
|  | Breast | Metastatic, any prior  therapy allowed | Olaparib | II | Olaparib, olaparib + atezolizumab | 72 | Recruiting | NCT02849496 |
|  | Ovarian, fallopian tube, peritoneal | Relapsed | Rucaparib | II | Bevacizumab + nivolumab + rucaparib | 76 | Recruiting | NCT02873962 |
|  | Ovarian | Maintenance following front-line platinum-based chemotherapy | Rucaparib | III | Rucaparib + nivolumab | 1000 | Active, not recruiting | NCT03522246 |
| Atezolizumab (MPDL3280A) | Ovarian | Platinum-sensitive, 2nd line + | Niraparib | I | Cobimetinib + niraparib, cobimetinib + niraparib + atezolizumab | 70 | Recruiting | NCT03695380 |
|  | Ovarian | Recurrent, no more than 2 prior lines chemotherapy | Niraparib | III | Platinum based regimens followed by maintenance niraparib, atezolizumab + platinum based regimens followed by maintenance niraparib + atezolizumab | 414 | Recruiting | NCT03598270 |
|  | TNBC | Germline BRCA 1/2- PD-L1+ metastatic TNBC | Talazoparib | II | Talazoparib + radiation (8 Gy x 3) + atezolizumab | 23 | Not yet recruiting | NCT04690855 |
|  | Urothelial | Metastatic, 2nd line+ | Niraparib | I/II | Atezolizumab, atezolizumab + enfortumab vedotin, atezolizumab + niraparib, atezolizumab + Hu5F9-G4, atezolizumab + isatuximab, atezolizumab + linagliptin, atezolizumab + tocilizumab | 305 | Recruiting | NCT03869190 |
|  | Pancreatic | Metastatic, 2nd line+ | Niraparib | I/II | Niraparib + nivolumab, niraparib + ipilimumab | 84 | Recruiting | NCT03404960 |
| Avelumab | Ovarian, breast, urothelial, prostate, NSCLC | Locally advanced, metastatic | Talazoparib | I/II | Avelumab + talazoparib | 214 | Active, not recruiting | NCT03330405 |
|  | Ovarian | Stage III or Stage IV, 1st line | Talazoparib | III | Avelumab + talazoparib | 79 | Active, not recruiting | NCT03642132 |
| Ipilimumab | Solid | Advanced, 2nd line+ | BGB-290 | I | BGB-A317 + BGB-290 | 230 | Recruiting | NCT02660034 |
| BGB-A317 | Solid | Advanced, 2nd line+ | BGB-290 | I | BGB-A317 + BGB-290 | 230 | Recruiting | NCT02660034 |
| Dostarlimab (TSR-042) | Ovarian | Stage III or IV, 1st line | Niraparib | III | Niraparib + dostarlimab | 1403 | Active, not recruiting | NCT03602859 |
| Abbreviations: ICB, immune checkpoint blockade; DDR, DNA damage repair; ER, estrogen receptor; HER2, human epidermal growth factor receptor 2; gBRCAm, germline BRCA mutated; SCLC, small cell lung cancer; NSCLC, non-small cell lung cancer; EGFR, epidermal growth factor receptor; ALK, anaplastic lymphoma kinase. | | | | | | | | |
